# Factors associated with malnutrition in children < 5 years in western Kenya: a hospital-based unmatched case control study

**DOI:** 10.1186/s40795-020-00357-4

**Published:** 2020-07-29

**Authors:** Edwin Gudu, Mark Obonyo, Victor Omballa, Elvis Oyugi, Cecilia Kiilu, Jane Githuku, Zeinab Gura, James Ransom

**Affiliations:** 1 Ministry of Health, Moi Teaching and Referral Hospital, Eldoret, Kenya; 2 Ministry of Health, Field Epidemiology & Laboratory Training Program, Nairobi, Kenya; 3 Center for Global Health Research, Kenya Medical Research Institute, Nairobi, Kenya; 4 West Pokot County Health Department, Kapenguria, West Pokot Kenya; 5 Ministry of Health, Division for Human Resource for Health Development, Nairobi, Kenya; 6 Piret Partners Consulting, 611 Pennsylvania Avenue SE, Unit 358, Washington, DC 20003 USA

## Abstract

**Background:**

Globally, under-nutrition accounts for > 3 million deaths annually among children < 5 years, with Kenya having ~ 35,000 deaths. This study aimed to identify factors associated with malnutrition in children aged < 5 years in western Kenya.

**Methods:**

We conducted a hospital-based unmatched case-control study between May and June 2017. Cases were defined as children aged 6–59 months with either z-score for weight-for-height ≤ −2SD or ≥ +2SD; weight-for-age ≤ −2SD or ≥ +2SD; or height-for-age ≤ −2SD. Controls were children aged 6–59 months with age-appropriate anthropometric measurements. Cases were consecutively recruited while systematic random sampling was used to select controls. Data from interviews and clinical records were collected and entered into Epi-Info, which was used to run unconditional logistic regression analyses.

**Results:**

A total of 94 cases and 281 controls were recruited. Of the cases, 84% (79/94) were under-nourished. Mother not having attended ante-natal clinic (OR = 7.9; 95% CI: 1.5–41.2), deworming (OR = 0.8; 95% CI: 0.4–1.2), and pre-lacteal feeding (OR = 1.8; 95% CI: 1.1–3.0) were associated with under-nutrition. Delayed developmental milestones (AOR = 13.9; 95% CI: 2.8–68.6); low birth weight (AOR = 3.3; 95% CI: 1.4–7.6), and paternal lack of formal education (AOR = 4.9; 95% CI: 1.3–18.9) were independently associated with under-nutrition.

**Conclusion:**

Proper pre-natal care, child feeding practices and deworming programs should be enhanced to reduce pediatric malnutrition.

## Background

Malnutrition refers to a state of either under-nutrition or over-nutrition. Under-nutrition occurs when the diet a person consumes does not meet their body’s requirement for growth and development whereas over-nutrition occurs when a person consumes too many calories [1]. Good nutrition and feeding practices are critical to a child’s growth and development especially during the first two years of life [2]. Under-nutrition impairs a child’s immunity, which can lead to recurrent infections, and impaired physical and cognitive development [3].

Under-nutrition is a major cause of morbidity and mortality especially, in low-to-middle-income (LMIC) countries. Globally, malnutrition contributes to more than 3 million deaths among children < 5 years annually [4]. UNICEF estimates that in Kenya, 239,446 children suffer from moderate acute malnutrition (MAM) and 2600 children suffer from severe acute malnutrition (SAM). Under-nutrition also contributes to about 35,000 deaths among children < 5 years each year in Kenya [4]. Stunting has also been linked to development of non-communicable diseases and lower adult productivity later in life. Children < 5 years who are prone to recurrent infectious diseases such as diarrheal illnesses, respiratory tract infections, tuberculosis and malaria often have under-nutrition as a co-morbidity [5].

The Kenya Demographic Health Survey 2014 reports that 26% of children < 5 years are stunted, 4% are wasted, and 11% are underweight. Malnutrition remains a public health concern in western Kenya. According to the survey 25.2% of children < 5 years are stunted while 8.2% are severely stunted [6]. This means that 1 in 4 children suffer from chronic under-nutrition. Therefore, identifying factors associated with malnutrition (especially under-nutrition) is vital in preventing the development of long-term deleterious effects.

This study aimed to identify clinical, demographic, and socio-economic factors associated with malnutrition in children < 5 years for public health action.

## Methods

### Study area

**Fig. 1:**
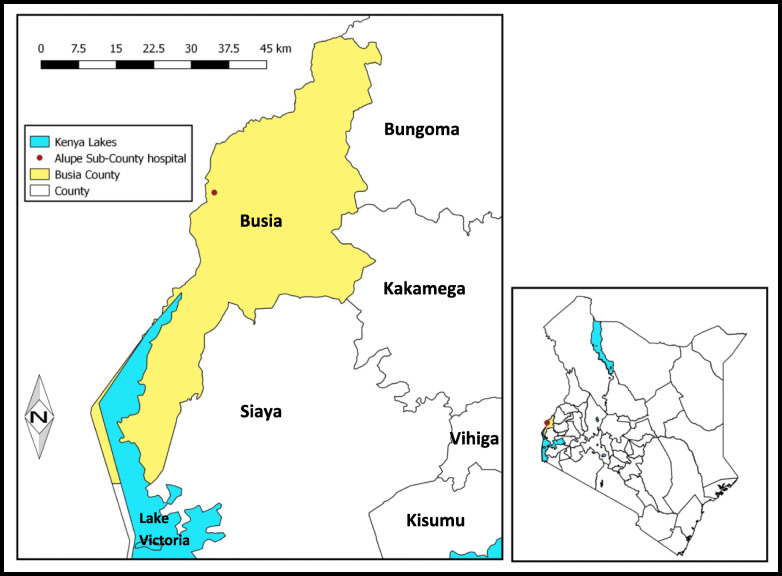
Map of Busia County, Western Kenya showing the constituent sub-counties including Teso South sub-county. Map source: Commission on Revenue Allocation-Kenya

The study was carried out at Alupe Sub-County Hospital. The hospital is a level 4 hospital located in Angorom ward, Teso South Sub-County in Busia County serving a catchment population of 34,321 persons (Fig. 1) [7].

### Study design

We conducted a facility-based unmatched case control study carried out between May 2017 and June 2017. We chose an unmatched design due to the more limited number of cases and the inconsistency and lack of some documentation of the data available in the records at the hospital. The study population consisted of all children < 5 years attending the child welfare clinic and the outpatient clinic within the hospital during the study period.

### Case definitions

Under-nourished child was defined as a child aged 6–59 months attending the hospital as an inpatient or outpatient whose anthropometric measurements were not appropriate for their age with z-scores (weight-for-height [WHZ], weight-for-age [WAZ], height-for-age [HAZ]) of <= − 2 SD. WAZ score from the WHO charts were used to define presence of under-nutrition [8].

A participant was classified as stunted if HAZ score was <−2SD and severely stunted if HAZ score was <−3SD. Wasting was defined as WHZ score < −2SD while severe wasting was WHZ score < −3SD. Any participant with WAZ score < −2SD was classified underweight.

### Mid upper arm circumference (MUAC) calculations

For the MUAC cut-points to determine whether a child was under- or over-nourished, we used the cut-points of any child with MUAC < 126 mm was classified under-nourished [8].

### Definition of controls

Any child aged 6–59 months attending the hospital as an inpatient or outpatient whose anthropometric measurements are appropriate for their age with z-scores between -2SD and + 2SD [9].

### Sample size determination

The sample size was calculated using statistical software Epi Info® version 7.2.0. The study assumed a 95% confidence interval, 80% power, 10% wasting among controls [10], and the ratio of cases to controls of 1:3. Using these assumptions, the minimum sample size was 375 (94 cases and 282 controls).

### Selection of cases and controls

The cases were sampled consecutively due to the low number seen each day for eligible children enrolled for nutritional support in welfare clinic. The sampling occurred via the data entered into the MoH Child Health Logbook, which would have each presenting child’s age, MUAC, and other information indicative of over-, under-, or at-level nutrition. The controls were selected through systematic random sampling from the data in the logbook. The average number of children < 5 years visiting the outpatient section of the child welfare clinic daily was used as a sampling frame. This was determined by obtaining the number of children visiting the out-patient clinic between April and June of three preceding years before the study. The study was conducted during weekdays within the duration of the study period hence the number of controls to be enrolled in the study on any single day was pre-determined. Using the average number of patients seen each day at the clinic and number of controls to be enrolled in the study each day, a sampling interval was determined, and the first control was picked randomly between one and the sampling interval. The sampling interval was then added to enroll the remaining controls. Any eligible participant whose legal parent/guardian did not give oral consent was replaced by the next available participant whose legal parent/guardian consented to the study.

### Data collection

Triage was carried out by the hospital staffs as is the norm and all critically and severely ill patients were urgently attended to by the hospital clinicians as per procedures and guidelines of the hospital. The weight was measured using electronic digital weighing scale (Seca®). For height/length, children < 2 years were measured lying down (recumbent length) while those who were > =2 years were measured standing up. For MUAC and head circumference, a non-stretch tape was used.

A pre-tested trans-adapted interviewer-administered questionnaire was used for each study participant to obtain demographic, clinical, nutritional, social and economic information. This questionnaire was adapted from a survey sheet used in Guinea [11]. (Each patient was de-identified by a unique code to ensure their privacy and maintenance of confidentiality.)

### Data management

Data entry, cleaning, validation and analysis was done using Microsoft Excel (Microsoft, Seattle, WA, USA), and Epi info version 7 (CDC, Atlanta, GA, USA). Anthropometric data was analyzed using WHO Anthro® software version 3.2.2 (WHO Anthro®) to assess nutritional indicators like weight-for-length/weight-for-height (wasting), weight-for-age (underweight or overweight), length-for-age/height-for-age (stunting), MUAC-for-age, and HC-for-age. The software then provided the z-scores based on gender, age and the anthropometric measurements. We calculated measures of central tendency and dispersion for the continuous variables and proportions for categorical variables. For univariable analysis, we calculated odds ratios (OR), 95% confidence intervals (CI), chi-square statistics and *p*-values. Variables with p-value ≤0.05 were statistically significant. We carried out unconditional logistic regression with variables that had p-values of < 0.2 at univariable analysis. A backward elimination stepwise method was used to identify independent factors associated with malnutrition. During model building, any variable that caused an insignificant increase in deviance on removal from the model were left out of the model while the variable that caused a significant increase in deviance on removal were retained in the model. All variables removed from the model when a backward stepwise method was performed and those known to be potential cofounders or factors associated with malnutrition from previous studies were tested for confounding, any of the mentioned variables that had a more than ten percentage change (> 10%) between the crude and adjusted odds ratio was considered as a confounder. The final model after testing for all biologically and statistically plausible interactions had only variables with *p*-value ≤0.05.

## Results

### Description of the study participants

There were 375 participants (94 cases and 281 controls), with median age of 16 months (IQR 10, 22), and 51% (191/375) male. Males were 57% (54/94) of cases and 49% (137/281) of controls.

### Nutritional status of cases

Of the cases, 84% (79/94) were under-nourished and 16% (15/94) over-nourished. Among those that were under-nourished, by assessing the WHZ score, 20% (16/79) were wasted while 9% (7/79) were severely wasted. Among the same group, using WAZ score, 39% (31/79) were underweight while 29% (23/79) were severely underweight. Using the HAZ score among the under-nourished, 46% (36/79) were stunted while 38% (30/79) were severely stunted.

### Univariable and multivariable analysis of factors associated with under-nutrition

On univariable analysis, socio-demographic factors like high birth order of five or more (OR = 2.3; 95% CI: 0.9–6.0), living in urban areas (OR = 1.9; 95% CI: 0.8–4.3), children whose mothers had no formal education (OR = 2.0; 95% CI 0.9–4.4), those whose fathers had no formal education (OR = 4.6; 95% CI: 1.4–15.0) and those who came from large family sizes of more than 6 occupants (OR = 1.8; 95% CI: 1.1–3.0) had higher odds of developing under-nutrition.

Pre-natal maternal factors were also shown to increase odds of developing under-nutrition. These included: participants whose mothers’ did not attend antenatal clinic (ANC) at least once (OR = 7.9; 95% CI: 1.5–41.6), participants whose mothers who did not attend 4 ANC visits as recommended by WHO (OR = 1.6; 95% CI: 0.9–2.7) and those whose mothers had illness during pregnancy (OR = 1.7; 95% CI: 1.0–2.8). The participants who were born preterm (OR = 2.0; 95% CI: 0.6–7.4) and those with low birth weight (OR = 2.8; 95% CI: 1.2–6.2) had higher odds of under-nutrition compared to term babies and those with normal birth weights.

Post-natal factors such as failure to complete or not being up-to-date on immunizations as per the national immunization schedule (OR = 2.2; 95% CI: 0.7–7.2) and human immunodeficiency virus (HIV) sero-exposure (OR = 1.4; 95% CI: 0.6–3.5) and delayed developmental milestones (OR = 18.9; 95% CI: 4.1–87.5) also increased the odds of developing under-nutrition. The participants who were eligible for deworming and had been dewormed at least once were protected from under-nutrition (OR = 0.8; 95% CI: 0.4–1.2). Infant and young child feeding practices also affected nutritional status of the participants. During the study period, 24% (89/375) of the participants were receiving pre-lacteal feeds increased their odds of under-nutrition (OR 1.8; 95% CI: 1.0–3.1). Exclusive breastfeeding for 6 months as recommended by the WHO was also widely practiced with 72% (271/375) of the participant’s parents adhering to this guideline. Forty percent (149/375) of the participant’s parents still used bottle with nipple for feeding, while 31% (116/375) ceased breastfeeding before the recommended 2 years of age. However, during the study period, there was no statistically significant association between duration of exclusive breastfeeding for the first six months of life (OR = 1.1; 95% CI 0.6–2.0), cessation of breastfeeding at less than 2 years (OR = 0.6; 95% CI 0.2–1.7) or bottle with nipple feeding (OR = 0.9; 95% CI 0.5–1.5) and developing under-nutrition.

Economic factors of the families also affected the nutritional status of the participants. Those whose mothers were unemployed had higher chances of under-nutrition (OR = 1.8; 95% CI 1.0–3.1) whereas those families with an average monthly income of above 5000 Kenya shillings (KES) were protective of under-nutrition (OR 0.7; 95% CI 0.4–1.2) (Table 1).

**Table 1:** Factors associated with under-nutrition, Alupe Sub-County Hospital, 2017^a^

Variable	Category	Cases ***n*** = 79 (***n***/%)	Controls ***n*** = 236 (***n***/%)	OR	95% CI	***P*** value
Residence	Urban	69 (87.3)	219 (92.7)	1.9	0.8–4.3	0.21
	Rural	10 (12.7)	17 (7.2)			
ANC attendance	Yes	5 (6.3)	2 (< 1)	7.9	1.5–41.6	0.02
	No	74 (93.7)	234 (99.1)			
Illness during pregnancy^1^	Yes	43 (54.4)	102 (43.2)	1.7	1.0–2.8	0.07
	No	33 (41.7)	129 (54.6)			
Gestation	Pre-term	4 (5.1)	6 (2.5)	2.0	0.6–7.4	0.46
	Term	75 (94.9)	230 (97.5)			
Low birth weight ^2^	Low	13 (16.5)	14 (5.9)	2.8	1.2–6.2	0.02
	Normal	57 (72.2)	169 (71.6)			
Delayed developmental milestones	Delayed	11 (13.9)	2 (< 1)	18.9	4.1–87.5	< 0.001
	Normal	68 (86.1)	234 (99.1)			
Deworming	Yes	49 (62.0)	163 (69.1)	0.7	0.4–1.2	0.31
	No	30 (38.0)	73 (30.9)			
Pre-lacteal feeds ^3^	Yes	25 (31.6)	50 (21.2)	1.7	1.0–3.1	0.08
	No	53 (67.1)	185 (78.4)			
Mother’s education level ^4^	None	11 (13.9)	18 (7.6)	2.0	0.9–4.4	0.14
	Other	67 (84.8)	218 (92.4)			
Father’s education level ^5^	None	7 (8.9)	5 (2.1)	4.6	1.4–15.0	0.02
	Other	69 (87.3)	227 (96.2)			
Average monthly income KES.	≥5000	25 (31.6)	96 (40.7)	0.7	0.4–1.2	0.20
	< 5000	54 (68.4)	140 (59.3)			
Birth order	≥5	8 (10.1)	11 (4.7)	2.3	0.9–6.0	0.14
	< 5	71 (89.9)	225 (95.3)			
Number of house occupants	≥6	41 (51.9)	88 (37.3)	1.8	1.1–3.0	0.03
	< 6	38 (48.1)	148 (62.7)			

^a^2 cases and 5 controls were brought by their guardians who did not know whether the child’s mother had any illness during pregnancy; 6 cases and 52 controls had high birth weight while 3 cases and 1 control were brought by guardians who did not know their birth weight*;* 1 case and 1 control had never been breastfed since birth (were on formula feeds)*;* 1 case was brought by a guardian who did not know the mother’s educational level*;* 3 cases and 4 controls were brought by guardians who did not know the father’s education levels

On multivariable analysis, delayed developmental milestones (AOR = 13.9; 95% CI: 2.8–68.6); low birth weight (AOR = 3.3; 95% CI: 1.4–7.6) and paternal lack of formal education (AOR = 4.9; 95% CI: 1.3–18.9) were found to be independently associated with under-nutrition.

## Discussion

The study identified various factors affecting nutritional status among children < 5 years which need to be adequately addressed. This included both pre-natal and post-natal factors as well as infant and young child feeding practices. Therefore, consistent follow-up of pregnant mothers from the antenatal period and post-natal care of the children < 5 years needs to be enhanced.

Among the undernourished, we found that stunting was the most common form of malnutrition, followed by children who were underweight and wasting being the least common among the study population. Stunting was common among cases of under-nutrition and over-nutrition alike. Stunting is a chronic form of malnutrition that results from prolonged non-adherence to proper dietary requirements to meet the body’s physiological needs. These findings were similar to those of a demographic and health survey carried out in the Western Kenya in 2014 [6]. Other studies carried out in Burundi and Uganda also had similar findings [12, 13].

Deworming of children > 1 year of age was also found to be protective of under-nutrition. This finding was in line with another study done in India among pre-school children which showed substantial weight gain among children who were dewormed [14]. This is because intestinal nematodes affect absorption of both micro and macronutrients which are vital for a child’s growth. However, current systematic reviews show little benefit is derived from mass deworming. They show that children found to be worm infested are the ones that gain weight more significantly compared to non-worm infested children [15, 16].

Proper breastfeeding practices for children are advocated for by WHO [17]. Children that are breastfed up to 2 years of age show quicker linear growth than those breastfed for shorter durations [2]. Feeding practices such as bottle with nipple feeding, breastfeeding within thirty minutes of delivery, exclusive breast feeding for 6 months and cessation of breastfeeding at 2 years were also assessed during the study. However, they were not statistically significantly associated with under-nutrition. In contrast, the giving of pre-lacteal feeds adversely affected nutritional status and predisposed the children to under-nutrition. This has also been shown by other studies [18–20]. This could be because pre-lacteal feeding affects the quality and quantity of breastfeeding which in turn affects the nutritional intake by the child. As such, proper education on feeding practices during post-natal period should be enhanced.

Children with under-nutrition were also shown to be more likely to have delayed developmental milestones. This finding was consistent with other studies [21–23]. This could be because they lack the macro and micronutrients necessary for normal growth and development. Children with prematurity and low birth weight also had higher odds of under-nutrition. These findings were similar from a review done in several countries [24]. This could be because they require more nutrients for catch-up growth which if not provided in adequate quantities leave them vulnerable to develop under-nutrition. These children should therefore be followed up more closely.

We also found that lack of parental formal education was linked to development of under-nutrition with paternal illiteracy being shown to have a greater influence. This finding concurred with other studies [20, 25]. This could be because the community being a patriarchal society, the fathers control the family’s resources. As such, lack of formal education could mean no formal employment and by extension no regular source of income to provide for their families.

Our study also showed that the cases of over-nutrition were also high, compared to findings of other studies in Kenya, despite the hospital serving a population of predominantly low socio-economic status [26]. This clearly points to the double burden of malnutrition that is supported by other literature based on a critical review done in other lower middle income countries [27]. This is a new development over the last couple of decades that needs to be further explored to halt and decrease the burden of cases of over-nutrition.

During the study period, children aged less than 12 months were more likely to be over-nourished. This finding was similar to another study carried out in Kenya in 2009 [28]. This could be because younger children are more likely to receive more attention and feeding effort from their parents as compared to older children. Male gender was also positively associated with over-nutrition. A study carried out in Kenya in 2016 had similar findings [29]. This could probably be due to the value and cultural preferences placed on the male child. As such, they are likely to be better fed as compared to the girl child. This has also been shown in other Sub-Saharan African countries [30].

Children who came from households in urban areas and those who came from families with higher average monthly income had higher odds of over-nutrition. This finding was similar to other studies [28]. This could be due to the higher levels of income which increase their ability to provide more than enough nutrition for their growing children.

Maternal lack of formal education also increased the chances of developing over-nutrition. This finding was contrary with other studies carried out in Sub-Saharan Africa [30]. High birth weight was also linked to increased chances of over-nutrition. This has also been shown by other studies [30, 31]. However, the exact mechanism of this link has not yet been clearly described.

We conducted a hospital-based case-control study and as such, its findings cannot be generalized to the entire population of under-five children in Western Kenya. The data collected on some of the variables could be susceptible to recall bias more so if the child was brought in by a guardian. Another limitation of the study was that the study relied on participants’ self-reported data, which was prone to recall bias and social desirability bias and interviewer bias due to the retrospective tracking of information beyond the advantages of case control study. The other limitation was that since it was a case–control study, which means it cannot establish the relationship between exposure and disease. Anthropometric measures and their technical errors are another limitation because it can result in misclassification of children’s nutritional status. However, we gave strict attention to the study procedures, including the process of training the research team and workers at the hospital, standardization of anthropometric measurements, and close and supportive supervision throughout the field activities to minimize biases.

## Conclusion

Proper pre-natal care, child feeding practices and deworming programs should be enhanced. As such, we recommend that close monitoring especially of children more likely to be malnourished should be enhanced. This can be done by providing job aids to providers to help them talk to parents about adherence to key recommended practices such as appropriate feeding, continuous auditing of patient outcomes, and better use of data for improved decision-making should be implemented at these facilities.

Proper infant and young child feeding practices and deworming should be emphasized. Provider advocacy and better health education to parents should be intensified in the region for better outcomes. The hospital in conjunction with Busia County Government should organize for regular outreach to the community targeting pregnant and lactating mothers, strengthen deworming programs for children > 1 year and all children with delayed developmental milestones. It should also organize for health advocacy camps targeting the parents with children < 5 years to educate them on the proper infant and young child feeding practices.

## Data Availability

All data generated or analyzed during this study are available upon request to the corresponding author.

## Abbreviations

MAM: Moderate Acute Malnutrition
MUAC: Mid Upper Arm Circumference
WHZ: Weight for Height Z-score
WAZ: Weight for Age Z-score
HAZ: Height for Age Z-score
SD: Standard Deviation
OR: Odds Ratio
AOR: Adjusted Odds Ratio
CI: Confidence Interval
